# Anatomical basis for Wilms tumor surgery

**DOI:** 10.4103/0971-9261.55151

**Published:** 2009

**Authors:** R. B. Tröbs

**Affiliations:** Department of Pediatric Surgery, Catholic Foundation Marien Hospital, Ruhr-University of Bochum, Herne, Germany

**Keywords:** Aorta, Gerota's fascia, Wilms tumor, inferior mesenteric artery, superior mesenteric artery, surgery, vascular injury

## Abstract

Wilms tumor surgery requires meticulous planning and sophisticated surgical technique. Detailed anatomical knowledge can facilitate the uneventful performance of tumor nephrectomy and cannot be replaced by advanced and sophisticated imaging techniques. We can define two main goals for surgery: (1) exact staging as well as (2) safe and complete resection of tumor without spillage. This review aims to review the anatomical basis for Wilms tumor surgery. It focuses on the surgical anatomy of retroperitoneal space, aorta, vena cava and their large branches with lymphatics. Types and management of vascular injuries are discussed.

## INTRODUCTION

The word surgery has Greek roots and means nothing more than “handwork.” This handwork is largely based on the science of dissection known as “anatomy.” From this point of view, pediatric surgery is anatomy applied to the living body of a child. The aim of my presentation is to review the anatomical basis of Wilms tumor (WT) surgery.

Treatment of children with a nephroblastoma is an interdisciplinary undertaking. In the last 40 years, attempts have been made to decrease the morbidity of treatment while maintaining an excellent survival rate. The pediatric surgeon or urologist plays a key role within the team. In particular, the duration and amount of chemotherapy and radiation therapy can be influenced by the surgical technique.

We can define two main goals for surgery (1) exact staging and (2) safe and complete resection of the tumor without spillage. The Société Internationale d'Oncologie Pédiatrique (SIOP) investigators pioneered the concept of pretreatment of renal tumors before nephrectomy. The SIOP trials showed that pre-treatment resulted in safer surgery, reduced tumor rupture rates, and an increased portion of children with a lower tumor stage. Thus, less overall treatment was required. These findings were confirmed by the Third Wilms Tumor (WT) Study of the United Kingdom Children's Cancer Study Group.[1,2]

Removal of a large nephroblastoma can still be a demanding undertaking. Large trials in WT surgery have identified various anatomy-based complications. However, institutional series and case reports give additional important details. Surgical problems in WT surgery can involve the staging and removal of the nephroblastoma itself, as well as hemorrhage and injuries to vessels and adjacent structures. The nomenclature of anatomical structures and eponyms used in this paper follows the recommendations of the Federative Committee on Anatomical Terminology.[3]

## ZONES, SPACES AND PLANES

Wilms Tumor is an embryonic renal tumor located within the retroperitoneal space. Recently, Skandalakis wrote “This space is a vast territory lacking any accurate knowledge and accepted map”.[4]

According Farthmann *et al*. (1989) the retro peritoneum can be divided into three zones and four parts: the central zone (containing the aorta, inferior vena cava, pancreas, and duodenum); two lateral zones (the kidneys, ureters, and ascending/descending colon), and the pelvic zone (rectosigmoid, iliac vessels, and urogenital organs).[4,5]

The retro-peritoneal space is defined as the space between the posterior parietal peritoneum and the transversalis fascia. This anatomic highway is responsible for the dissemination of pathologic entities originating in the retro peritoneum, and is comprised of connective tissue layers.

Gerota's fascia is often used as a general term to describe renal fascia. In 1883, Zuckerkandl described the posterior renal fascia but did not recognize the presence of the anterior layer of renal fascia. In his work Beiträge zur Kenntnis des Befestigungsapparates der Niere, the Romanian Gerota documented the presence of the anterior fascia and clearly assigned Zuckerkandl's name to the posterior fascia.[6]

**Figure 1:**
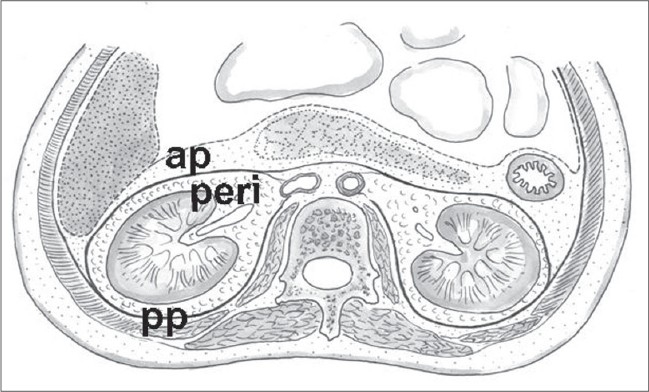
Peri- and pararenal compartments (ap – Anterior, pp – Posterior Pararenal)

Three compartments of retro peritoneal space are related to the kidney: the perirenal space as well as the anterior and posterior pararenal spaces [Figure 1]. The perirenal space is the home of the kidneys. The renal fascia, a collagenous connective tissue of mesodermal origin that envelopes the kidney, is responsible for this compartmentalization. The kidney is enveloped by the anterior and posterior laminae of the renal fascia and fatty tissue inside and outside the fascia. There is some medial fixation with the adventitial covering of renal vessels and aorta or inferior vena cava (IVC).[4]

**Figure 2:**
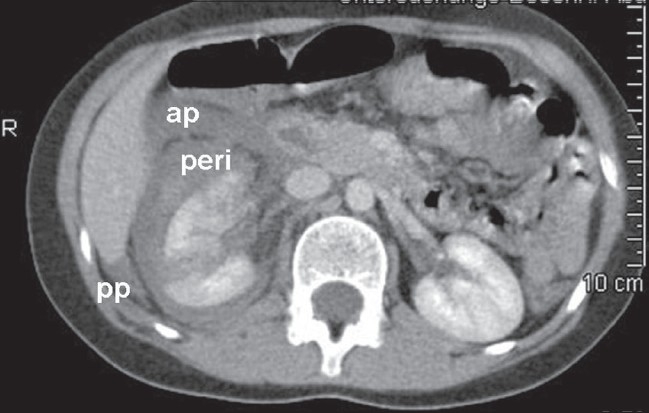
Peri- and pararenal hematoma after traumatic renal rupture (CT Scan)

**Figure 3:**
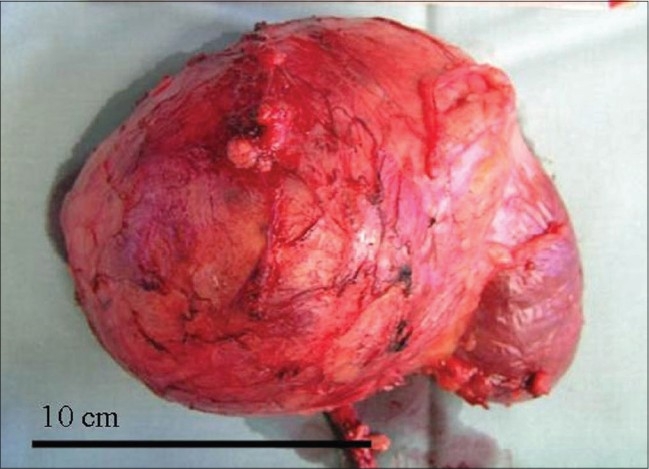
Nephrectomy specimen. Upper pole tumor covered by Gerota's fascia

For example, Figure 2 depicts a case of traumatic renal rupture. The distribution of perirenal blood and urine clearly shows the existence of three renal compartments in a child. The ruptured right lower pole is shown with separated blood collections in the perirenal space (asterisks) as well as in both pararenal spaces (stars). The rate of perirenal infiltration of nephroblastomas remains unclear. To obtain clear margins, a rim of healthy tissue including covering fibrous tissue and fat has to be resected with the tumor. Strict orientation between different retroperitoneal planes is required to achieve this. Standard textbooks recommend that the tumor plane should be developed outside the perirenal fascia.[7–10] In other words, resection of the tumor-bearing kidney during radical uretero-nephrectomy also requires removal of the intact Gerota's and Zuckerkandl's fascia that cover the kidney. This procedure is known as a perifascial nephrectomy. The uretero-nephrectomy specimen shown in Figure 3 demonstrates an upper pole tumor removed with a sheath of surrounding fat and perirenal fascia.

At the upper pole of the kidney, a fascial septum separates the adrenal gland from the kidney.[4] The adrenal gland for small or lower pole tumor can be spared.[9] However, in many cases, when the tumor is large and adrenal gland is attached it must be removed to achieve adequate margins.

## VESSELS – KEYS TO SUCCESSFUL SURGERY

Generally, three types of vessels have to be taken into account: arteries, veins, and lymphatic vessels. Modern imaging techniques allow clear outlining of many aspects of the visceral blood supply. The aorta and its branches are quite small in infants and toddlers; thus they can be mistaken for the renal artery. In a three-year-old child the aorta at the level of the kidneys has a diameter of 6 to 7 mm and the renal artery measures 2.8 mm.[11] WT surgery is a safe and partially standardized procedure. However, major bleeding and vascular injuries can occur. The frequency of vascular injuries has been reported as 1.5% in the NWTS-3 and 4.[12,13] Severe hemorrhage occurs at a higher rate. Further, there exists a hidden surgical mortality.[14]

The vascular pedicle of both kidneys is an important anatomical area and should be analyzed. The left renal vein passes in front of the aorta in the majority of individuals. The right artery reaches the kidney behind the IVC. However, variations in surgical importance can occur. In up to three per cent cases, a retro aortic left renal vein has to be taken into account. Circum aortic veins also occur in a relevant number of patients.[15]

Ligature and cutting of the large renal vessels is one of the most important steps of nephrectomy. Early ligature of the vein has the theoretical advantage of preventing hematogenous tumor spread.[8,16,17] However, this was never confirmed by a prospective trial. On the other hand, primary ligature of the artery is recommended for prevention of tumor swelling and rupture.[7,10,18]

There is no consensus in literature regarding the sequence of vessel ligature. Selected international standard publications recommend early control of the hilum. However, this is often not feasible with extremely large tumors; mobilization of the tumor mass must first occur to allow exposure of the hilar vessels.[16,19] The current SIOP 2001/German Society of Pediatric oncology protocol recommends initial ligature of the artery. When feasible, we follow this recommendation.

During removal of left renal tumors, damage to the aorta, superior mesenteric artery (SMA), and right renal artery has been reported to occur. These vessels are in close proximity to the tumor mass, and if the aorta and IVC separate by tumor or lymphatic infiltration, they are threatened during removal of the left kidney. The left renal vein is usually identified first. Once it is divided another artery is revealed underneath that could be the superior mesenteric artery, aorta, or left or right renal artery. This artery should not be ligated until its exact identity has been established.[20,21] When in doubt, this can be done by cross-clamping the vessel with a vascular clamp. It can be clearly stated that attempts at early ligation of the hilar vessels cannot be justified until the renal vasculature is clearly identified.

The vessels most at risk during excision of right renal tumors are IVC and contralateral renal vein. In large right-sided tumors, the IVC enters the tumor mass and is hidden from vision. Under this circumstance, the right renal artery is in close proximity to the left renal vein, which can thus be damaged.[20] If unrecognized, such damage can lead to venous infarction and loss of renal function.

## AORTA AND ITS BRANCHES

**Figure 4:**
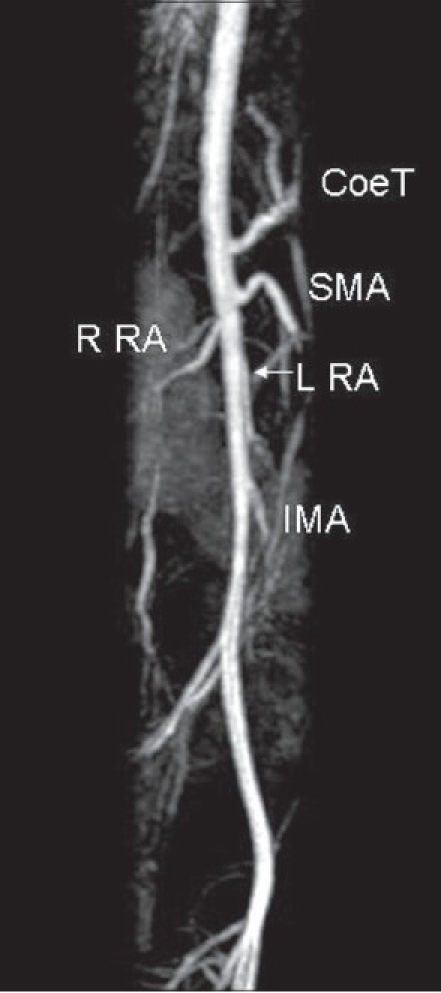
Major Branches of Abdominal Aorta (MRI). R RA – Right Renal Artery, L RA - Left Renal Artery

Modern imaging techniques allow noninvasive clear outlining of the visceral blood supply. The angio-MR [Figure 4] demonstrates the arterial branches of the aorta in a child with a left-sided WT.

It shows that the coeliac trunk (Coe T) and SMA originate from the aorta in close proximity to the origins of the renal arteries. The distance between the origins of these aortal tributaries can be 1 cm or less.[22]

**Figure 5:**
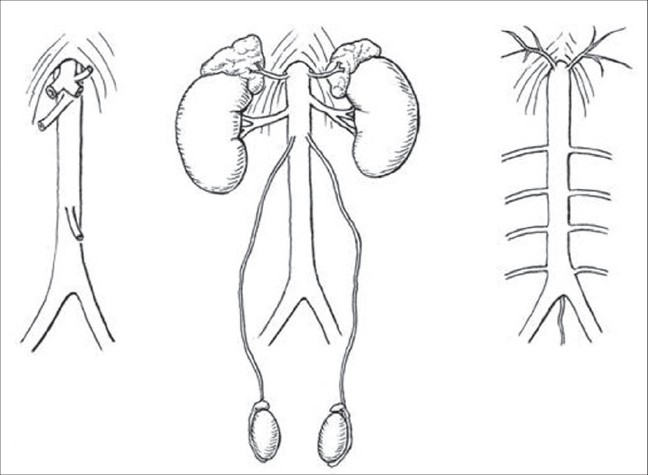
Three Planes of Aortic Branches.[4]

For surgical practice it is helpful to arrange the aortic branches in the three planes they occupy [Figure 5]: (1) blood supply to the gastrointestinal tract in front of the aorta; (2) to three paired glands on both sides and (3) to diaphragm and the four lumbar arteries.[4]

Iatrogenic injury to the aorta and its branches has been inconstantly reported. These reports indicate that patients with left sided large tumors are at particular risk for this type of injury. Although attempts were always made to repair the intraoperative vascular injury, three of six patients described in literature died as a result of the vessel injury.[23,24] All these children had left sided nephroblastoma; four cases had injuries to the SMA, one had both SMA and coeliac trunk, and one the aorta injury. It has been reported that after unnoticed ligation of SMA, though the bowel may initially appear viable, full thickness necrosis develops later. When in doubt, a Doppler flow study can provide essential information.

Lacerations of the SMA require surgical repair.[23,25] Fullen and coworkers attempted to classify SMA injuries, and found that at least injuries proximal of the middle colic artery urgently require repair, whereas those of the distal part can be tolerated without ischemia or with ischemia of only a small segment of the bowel.[25] When possible, end-to-end anastomosis of the vessel with or without venous interposition or end-to-side anastomosis to the aorta is the procedure of choice.

**Figure 6:**
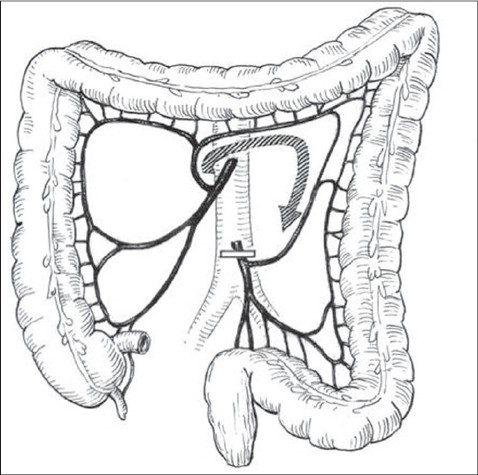
Marginal Arcade and AALCA Connect SMA with IMA.[26,27]

In contrast to SMA, dissection of the stem of inferior mesenteric artery (IMA) is commonly tolerated without disastrous consequences. Generally the marginal artery, also known as arcade of Riolan arch, (synonymously marginal artery of Drummond), sufficiently connects SMA with IMA [Figure 6]. However, only small collaterals exist at the splenic flexure.[22] In addition, the ascending arch of the left colic artery (AALCA) is present in at least two third of cases. This arterial branch constitutes an arch from the left transverse colon to the sigmoid colon secondary to the marginal artery.[26,27]

## VENOUS DRAINAGE

**Figure 7:**
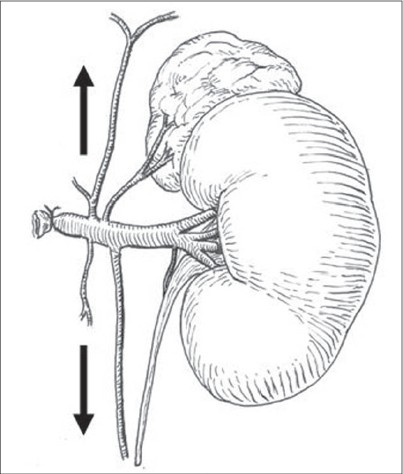
Venous Drainage of Right Kidney

The left and right kidneys show a difference in venous drainage. In right-sided nephroblastoma with cava thrombus, ligature and dissection of the left renal vein is possible. In the majority of patients, sufficient venous collaterals via the phrenic, adrenal, hemiazygos, testicular, lumbal and ureteral veins are present [Figure 7].[28] In contrast, the anatomy of the right kidney does not allow this maneuver.[29]

In humans, embryogenesis of the renal and post renal segments of IVC involves the sequential appearance of three paired venous channels: the posterior cardinals as well as the sub cardinal and supra cardinal veins.[30] The development of anastomotic channels between these channels and subsequent regression of segments of this system might be impaired, leading to variants like the persistence of the left sub cardinal vein leading to a high confluence of the large veins. Other rare anomalies of the IVC include duplication, left-sidedness of the vein and interruption of the IVC with azygos/ hemiazygos continuation.[15,30,31]

Lack of recognition of retro peritoneal venous anomalies can have potentially disastrous consequences because anomalous venous structures tend to be dilated and tortuous. Intraoperative lacerations can cause life-threatening hemorrhage.

## LYMPH NODES AND VESSELS

Surgeons might not be able to tell from gross inspection whether or not lymph nodes contain tumors.[32] Random sampling of nodes in each area should be performed since occult lymph node metastases affect the tumor stage and therapy. Failure to sample lymph nodes results in a poorer prognosis. Postoperative chylous ascites has been infrequently described after WT nephrectomy.[33,34] The usual causes are radical lymphadenectomy and operative injury to the cisterna chyli or their major tributaries. Resection of the supra hilar nodes carries a particular risk of injuring the cisterna. Meticulous lymphostasis is of paramount importance in such cases of lymph node removal.
